# Supplementary material for: Prognostic Implication of Ventricular Volumetry in Early Brain Computed Tomography after Cardiac Arrest
Source: Diagnostics (Basel). 2024 Aug 6;14(16):1701. doi: 10.3390/diagnostics14161701 (PMC11353943; doi:10.3390/diagnostics14161701)
Supplement: Supplementary file 1 [file diagnostics-14-01701-s001.zip › diagnostics-3136297-supplementary.pdf]

Supplementary Materials

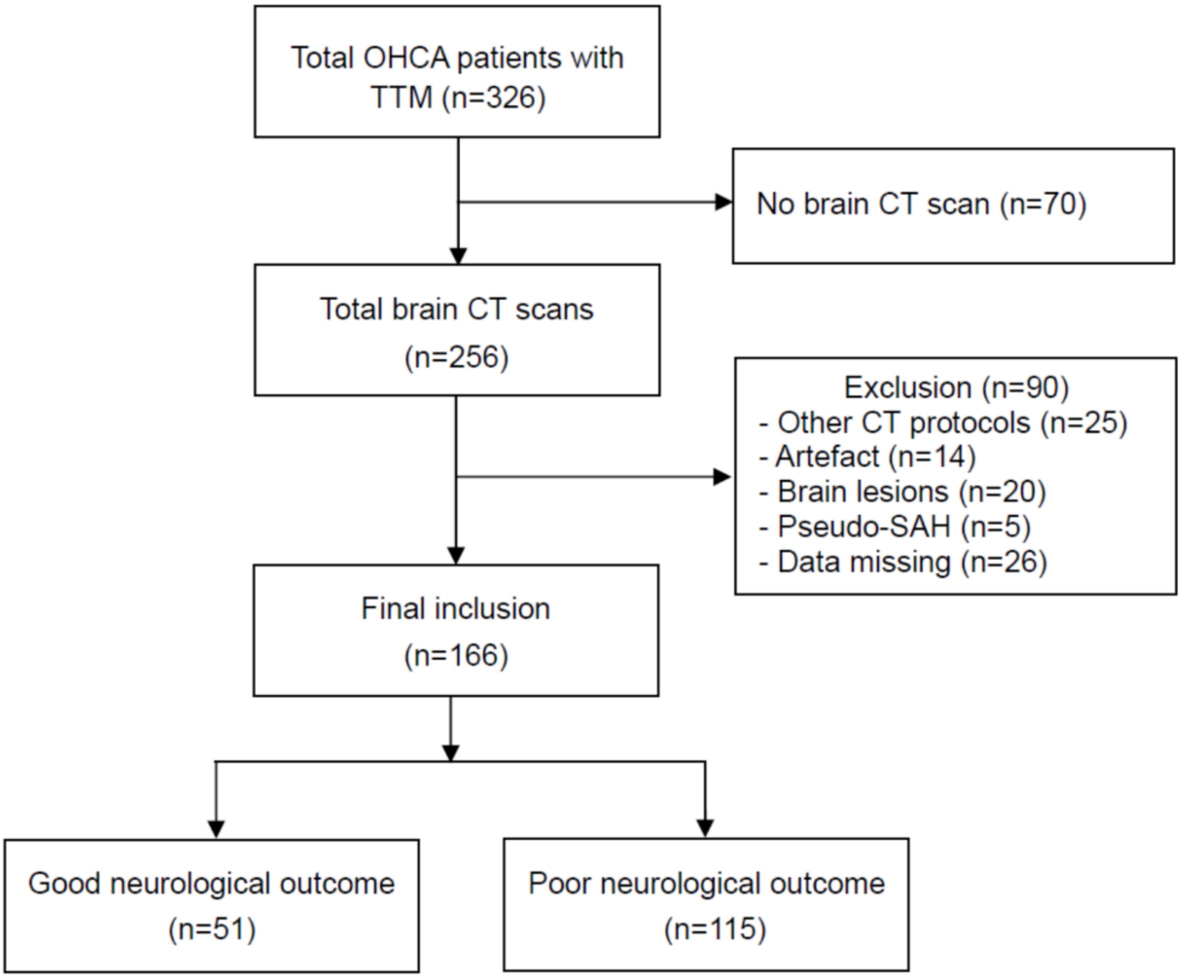

Figure S1. Flowchart showing the patients included in this study.

OHCA, out-of-hospital cardiac arrest; TTM, targeted temperature management; CT, computed tomography; SAH, subarachnoid hemorrhage

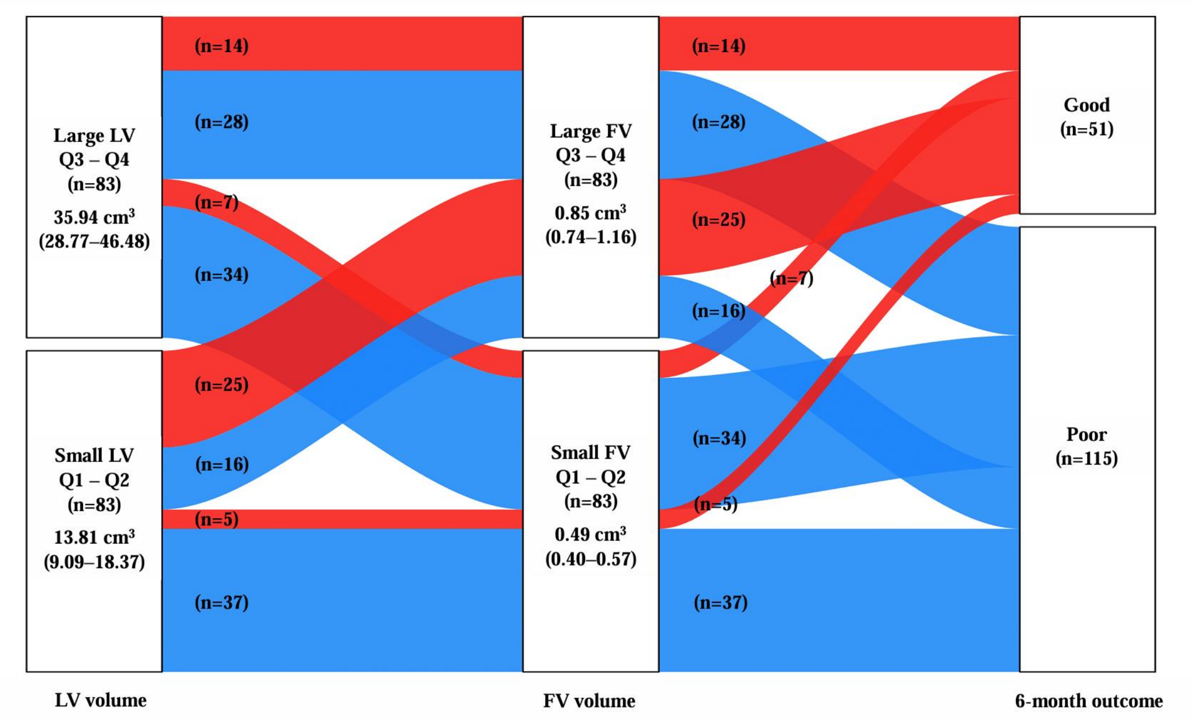

Figure S2. Alluvial plot with subjects from the good and poor neurological outcome groups divided into quartiles (Q1-Q4) based on the volumes of the lateral and fourth ventricles. The median (IQR) ventricle volumes are presented for each quartile. Alluvial plots show how each lateral ventricular volume quartile is distributed over the volume of the fourth ventricle based on the 6-month neurological outcome (colored red and blue).

Table S1. The intraclass correlation coefficients.

	intraclass correlation coefficient	P
Lateral ventricular volume	0.88	< 0.001
Third ventricular volume	0.96	< 0.001
Fourth ventricular volume	0.85	< 0.001
